# Prolyl Oligopeptidase, Inositol Phosphate Signalling and Lithium Sensitivity

**DOI:** 10.2174/187152711794653779

**Published:** 2011-05

**Authors:** Adrian J Harwood

**Affiliations:** School of Biosciences, Cardiff University, Museum Ave, Cardiff, CF10 3AX, UK

**Keywords:** Bipolar mood disorder, *Dictyostelium*, inositol phosphate signalling, lithium, multiple inositol polyphosphate phosphatase, prolyl oligopeptidase.

## Abstract

Inhibition of prolyl oligopeptidase (PO) elevates inositol phosphate (IP) signalling and reduces cell sensitivity to lithium (Li^+^). This review discusses recent evidence that shows PO acts *via* the multiple inositol polyphosphate phosphatase (MIPP) to regulate gene expression. As a consequence, PO inhibition causes both a transient, rapid increase in I(1,4,5)P_3_ and a long-term elevation of IP signalling. This pathway is evolutionary conserved, being present in both the social amoeba *Dictyostelium* and human cell systems, and has potential implications for mental health.

## INTRODUCTION

An intriguing aspect of prolyl oligopeptidase (PO) biology is its relationship with bipolar mood disorder and lithium (Li^+^) sensitivity. Bipolar mood disorder, also know as manic-depression, is a prevalent mental illness with a significant socio-economic impact. The biological causes of this major mental disorder remain unknown and, despite a strong genetic evidence for predisposition, there are currently no universally agreed consensus candidate genes that confer risk. Li^+^ is the most commonly used mood stabilizer used in the treatment of bipolar mood disorder and is particularly useful as a prophylactic, suppressing the re-occurrence of the illness. However, Li^+^ is taken in high doses, has side-effects and is not effective in all cases. Although a number of enzyme targets have been well characterised, how Li^+^ treatment mediates its action also remains unknown. This review describes recent results investigating how PO modulates cell sensitivity to Li^+^, and how this may relate to the treatment and molecular origins of bipolar mood disorder.

## BIOCHEMICAL TARGETS OF LITHIUM

### Inositol Phosphate (IP) Signalling

**Fig. (1):**
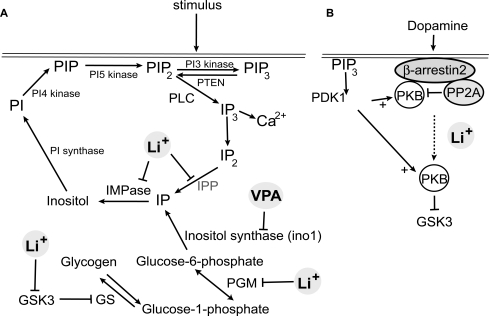
**Cellular targets of Li^+^.** (**A**) Inositol phosphate (IP) biosynthesis and signalling. Inositol is recycled from I(1,4,5)P_3_ (IP_3_) by inositol polyphosphate 1-phosphatase (IPP) and inositol monophosphatase (IMPase). It is also synthesised from glucose-6-phosphate *via* inositol synthase to inositol monophosphate (IP). Glucose is released from glycogen in the form of glucose-1-phosphate, which is then converted to glucose-6-phosphate by phosphoglucomutase (PGM). Glucose is incorporated into glycogen, *via* glucose-1-phospahte and UDP-glucose intermediates, by Glycogen synthase (GS), which in turn is inhibited by glycogen synthase kinase 3 (GSK3). Inositol is incorporated into PI(4,5)P_2_ (PIP_2_) by PI synthase, PI4 kinases and PI5 kinases. Upon cell stimulation, PIP_2_ is either converted to PIP_3_ *via* activation of PI_3_ kinase or is hydrolysed to release IP_3_, which in turn stimulates calcium release from intracellular stores. PIP_3_ is dephosphorylated to PIP_2_ by PTEN, which requires PIP_2_ to associate with the plasma membrane. Li^+^ inhibits IMPase, IPP, PGM and GSK3, whereas valproic acid (VPA) inhibits inositol synthase. All of these targets have the potential to reduce myo-inositol. GSK-3 has many other cellular targets (not shown). (**B**) Dopamine and regulation of PKB. PIP_3_ stimulates activation of the protein kinase, PKB, whereas dopamine suppresses PKB activity by formation of a complex between β-arrestin2, PKB and the phosphatase PP2A. Li^+^ causes the β-arrestin2 complex to disassemble, increasing PKB activity, which then phosphorylates and inactivates GSK-3.

The first enzyme target of Li^+^ identified was inositol monophosphatase (IMPase) [1]. This dephosphorylates inositol monophosphate to release myo-inositol, which is then transferred to diacyl glycerol to form phosphatidyl inositol (PI). This is then further phosphorylated on the inositol ring to create a range of lipid molecules with various signalling functions (Fig. **1A**). The two most studied are PI(4,5)P_2_ and PI(3,4,5)P_3_ (PIP_3_) [2, 3] although other lipids, such as PI(3)P and PI(3,4)P_2_ are important in membrane trafficking in the cell [4]. Inositol monophosphate is formed by dephosphorylation of the soluble inositol phosphate, I(1,4,5)P_3_ (IP_3_) by specific IP_3_ 5’phosphatases to I(1,4)P_2_ and then further dephosphorylation by inositol 1-polyphosphate phosphatase (IPP), a second lithium sensitive enzyme with a protein structure closely related to IMPase [5] (Fig. **1A**). Alternatively myo-inositol is synthesised *de novo* by isomerisation of glucose-6-phosphate through the action of inositol synthase (Ino1) [6]. As a consequence cellular synthesis of myo-inositol is sensitive to Li^+^. This has the potential to deplete the cellular concentration of inositol, however in many cases inositol can be taken up from the environment *via *transporter proteins in the membrane [6]. A significant change in IP signalling due to inositol depletion presumably occurs in cells where environmental inositol is low, however in some conditions Li^+^ and other mood stabilizers may also lower inositol up-take potentially enhancing their effects [7].

PI(4,5)P_2_ is a substrate for the enzyme phospholipase C (PLC), which, following cell stimulation, releases the second messenger, IP_3_. This binds the IP_3_ receptor on the endoplasmic reticulum to release Ca^2+^ from its intracellular store [8]. This transient elevation of intracellular Ca^2+^ elicits a range of cellular processes, including cell motility, cell survival and neurotransmitter release. Li^+^ is well documented in lowering IP_3_ and cellular Ca^2+^ levels in cultured cells, however its effects on the brain tissue can vary on cell type or species [9], and may reflect variation of indirect effects though feedback from downstream processes, such as glutamate uptake [10].

However, there are other inositol containing signal molecules in the cell, and a particular interesting molecule is the phospholipid, PIP_3_. This is synthesised from PI(4,5)P_2_ following stimulation of class 1 PI3 kinases (PI3Ks). PIP_3_ is also an important cell regulator being involved in many processes including cell growth, survival and chemotaxis [11]. Once generated on the plasma membrane PIP_3 _acts as a binding site for PH domain proteins, bringing them in contact with activator and effector proteins. For example, PIP_3_ promotes contact between the protein kinases  and PKB (also known as AKT) allowing  phosphorylation and activation of PKB [12, 13].

**Fig. (2):**
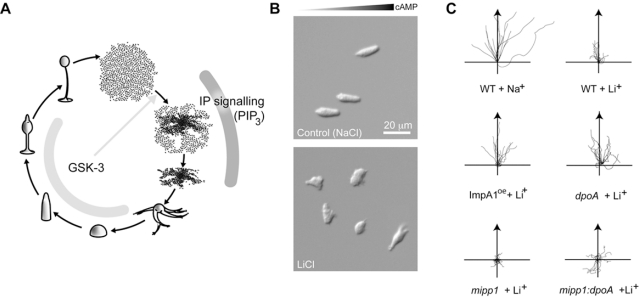
**Li^+^ sensitivity of *Dictyostelium* of development and chemotaxis.** (**A**) *Dictyostelium* developmental cycle. Individual cells enter development upon starvation and aggregate *via* chemotaxis to form a mound of cells. The mound undergoes a series of morphogenetic changes to give rise to the fruiting body. When dispersed, spores germinate and grow as amoebae. There are a number of Li^+^-sensitive stages of *Dictyostelium* development. The Li^+^-sensitive protein kinase, GSK-3, regulates cell fate during multicellular development and acts at the beginning of development as a permissive signal to allow chemotaxis and cell signaling (grey arrow). During aggregation, Li^+^ has a distinct role mediating inositol phosphate signaling and is required for PIP_3_ signalling. (**B**) Li^+^ alters chemotaxis. Photographs of wild type cells treated with LiCl or NaCl (control) during chemotaxis towards cAMP. (**C**) Tracks of representative cells migrating towards a cAMP source. Wild type cells treated with 7 mM Li^+^ or Na^+^ (control). Li^+^ treated cells move towards the cAMP source but move with half the speed and turn more than control cells. Increased expression of IMPase or loss of DpoA suppresses this effect; where as loss of MIPP enhances this effect. Based on original data published in [35].

Surprisingly, until recently the notion that PIP_3 _could be sensitive to Li^+^ had not been tested. However, a direct effect of Li^+^ on PIP_3 _has recently been shown in the social amoeba *Dictyostelium *[14]. *Dictyostelium* is a single celled eukaryote with a close phylogenetic relationship to the animals. It grows as unicellular amoeba, but when depleted of nutrients enters a developmental program to form a multicellular fruiting body [15] (Fig. **2A**). This is a highly differentiated structure comprising a small number of cell types. The majority of cells differentiate into spore cells, which are resistant to environmental stress, but germinate to release amoeba when conditions favour cell growth. The remaining cell types form the fruiting body structure that supports the spore head. Early *Dictyostelium* development is dependent on chemotaxis towards extracellular pulses of cAMP, which brings cells together into the multicellular aggregation. PIP_3_ based signalling is an important part of this process and is sensitive to Li^+^ (Fig. **2A**).

Li^+^ treatment of *Dictyostelium* cells has a specific effect on cAMP-dependent chemotaxis, reducing cell speed and path linearity, but having only a minor effect on the ability to sense the direction of the cAMP source (Fig. **2B**, **C**). Exactly the same effects are seen in mutants that lack all five *Dictyostelium* PI3Ks, or cells treated with the PI3K inhibitor LY294002 [16, 17]. Measuring either absolute PIP_3_ concentrations, phosphorylation of PKB or translocation of a GFP-fused PH domain showed that Li^+^ indeed suppresses PIP_3_ synthesis following cAMP stimulation [14]. Similarly, PIP_3_ signalling is also suppressed in the human HL60 neutrophil-like cell line following stimulation with the ligand fMLP. Consistent with inositol depletion, over-expression of IMPase in *Dictyostelium* cells reverses the effects of Li^+^.

Interestingly, in the absence of Li^+^, IMPase over-expressing cells show higher levels of stimulated PIP_3_ than wild type cells, indicating that inositol synthesis is limiting for cell signalling. This comes as a surprise as the steady-state concentration of PI(4,5)P_2_ is 200 times that of PIP_3_ and is unaltered by Li^+^ treatment. However, when newly synthesised PI(4,5)P_2_ is measured, Li^+^ causes a 60% decrease that mirrors that of PIP_3_. Over-expression of IMPase reverses the effect of Li^+^, whereas PI(4,5)P_2_ concentrations are higher than wild type cells in the absence of Li^+^ [14]. These results indicate that there is a rapidly synthesised, but small, pool of PI(4,5)P_2_ that is sensitive to Li^+^ treatment.

### Glycogen Synthase Kinase-3 (GSK-3)

The second major Li^+^ target is GSK-3, a highly conserved protein kinase which is involved in a plethora of cellular processes, including differentiation and development, growth and survival, chemotaxis and metabolism, as well as controlling glycogen synthesis [18]. As a consequence, mis-regulation of GSK-3 has been associated with a number of clinical conditions, such as diabetes, oncogenesis and neurodegeneration. Li^+^ inhibits GSK-3 with a Ki of 2.0 mM for the GSK-3β isoform and an IC_50_ of 3.5 mM for the GSK-3α isoform [19, 20]. Biochemical analysis showed that maximal GSK-3 activity requires high concentrations (~20 mM) of Mg^2+^, and that this Mg^2+^ dependency is competed by Li^+^. As Li^+^ is a non-competitive inhibitor with regard to its substrate and ATP, it would appear that the Li^+^ sensitivity of GSK-3 is *via *a Mg^2+^-binding site that is distinct from the Mg-ATP binding site present in all other kinases [21]. Consistent with this notion, GSK-3 is the only known Li^+^ sensitive protein kinase.

### Other Li^+^ Targets

A third enzyme target of Li^+^ is phosphoglucomutase (PGM) [22]. This enzyme reversibly converts glucose-1-phosphate to glucose-6-phosphate, and is inhibited in the same concentration range of Li^+^ as IMPase and GSK-3. Interestingly, GSK-3 is a negative regulator of glycogen synthase, reducing glucose conversion to glycogen, and hence Li^+^ inhibition of GSK-3 would lead to decreased glucose. As glucose-6-phosphate is the substrate for Ino1, inositol synthesis would be expected to be sensitive to altered activity of both PGM and GSK-3. This suggests that Li^+^ treatment could potentially be able to reduce inositol biosynthesis *via *simultaneously inhibiting all three enzyme targets (Fig. **1A**).

In contrast, Li^+^ has also been suggested to have a non-enzymatic action by disruption of a protein complex containing β-arrestin2, the phosphatase PP2A and PKB [23]. The complex forms following dopamine binding to its D2 receptors, leading to inactivation of PKB due to dephosphorylation by PP2A. Li^+^ treatment releases PKB from the complex increasing its activity, and in turn phosphorylating and inactivating GSK-3 (Fig. **1B**). Exactly how this works is unclear, and in fact it has frequently been reported that inhibition of GSK-3 feeds back to elevate its own phosphorylation *via *PKB [24-26], so it remains to be conclusively established whether this mechanism is truly independent of GSK-3 inhibition.

## LI^+^ SENSITIVITY MUTANTS OF *DICTYOSTELIUM*

Li^+^ has a variety of effects on *Dictyostelium* development, which can be distinguished by dose and timing. In addition to its effect on chemotaxis and PIP_3_ signalling, lower concentrations of Li^+^ cause altered multicellular development, where the one population of stalk cells enlarges at the expense of the spore head [27]. This is due to a change in cell fate during early multicellular development due to blocking the regulatory effects of cAMP on cell differentiation, and closely resembles the phenotype seen when the *Dictyostelium* GSK-3 gene is mutated [28]. GSK-3 is also required at the very onset of *Dictyostelium* development and GSK-3 mutants are incapable of both cAMP signalling and chemotaxis [29] (Fig. **2A**). However loss of GSK-3 activity has very different effects from the chemotaxis deficit that arises due to suppression of PIP_3_ signalling [14] and studies in* Dictyostelium* offer an easy means of distinguishing between different lithium targets on the basis of altered cell behaviour, something that is often difficult with other cell systems.

Using resistance to Li^+^ as a phenotype, a series of mutants with reduced sensitivity were isolated; these were referred to as *lis* mutants [27] (Fig. **2C**). The first mutant to be fully characterised was *lisA*. This mutant arose from an insertion into the *Dictyostelium* PO gene, DpoA, and lacked all PO activity. Importantly, no suppression of the GSK-3 mutant phenotype was seen when GSK-3 and DpoA mutations were combined. However, loss of *dpoA* or treatment with PO inhibitors elevates IP_3_, suggesting that PO is a modulator of IP signalling [27].

## PO AND MULTIPLE INOSITOL POLYPHOSPHATE PHOSPHATASE (MIPP)

Given the known action of PO on neuropeptides and peptide hormones [30], initial thoughts were that decreased extracellular peptide signals could lead to higher IP_3_ through chronic PLC stimulation. However a number of observations argue against this possibility. First, there is no evidence for increased PLC activity following loss or inhibition of DpoA [27]. Second, phenotypes of PLC and IP_3_ receptor mutants do not match the PO phenotype [31, 32]. Finally, PO activity is cytosolic and would not directly act on an extracellular signal peptide [27]. Consistent with this, experiments of mixing wild type and DpoA mutant cells demonstrated that the signalling defect is cell non-autonomous [27]. If IP_3_ is not generated by activation of PLC, could it be due to decreased IP_3_ breakdown by an IP_3_ 5’ phosphatase? In fact, 5’ phosphatase activity is increased rather than decreased in DpoA mutant cells, arguing that the production of IP_3_ is actually higher than measured by steady state levels of IP_3_ in the mutant [27]. These observations exclude PLC and 5’phosphatase activities as the source of IP_3_.

Multiple inositol polyphosphate phosphatase (MIPP) is a histidine acid phosphatase that dephosphorylates the higher order inositol phosphate IP_6_ to IP_3_ *via *IP_5_ and IP_4_ intermediates [33-35]. It also can dephosphorylate 2,3-diphosphoglycerate (DPG) and offering a new addition to the Rapport-Lubering shunt, a side branch of glycolysis [36]. Without 2,3 DPG, oxygen would bind too tightly to haemoglobin [37, 38], and as MIPP activity is sensitive to changes in over a pH range of 7.0-7.8, it is ideally suited to regulate oxygen binding in response to decreased carbon dioxide.

Mutants lacking DpoA have decreased MIPP activity, leading to increased IP_3_ [27], whereas those lacking the MIPP gene do not increase IP_3_ following PO inhibition [35]. This suggests that DpoA acts to represses MIPP activity altering the cellular IP_3_ concentrations. Consistent with these observations, MIPP mutants are Li^+^ hypersensitive, showing a significantly stronger effect of Li^+^ on chemotaxis (Fig. **2C**). Mutants that combine both DpoA and MIPP mutations are Li^+^ hypersensitive, not resistant, placing MIPP genetically downstream of DpoA [35].

The DpoA-MIPP interaction can be reconstituted in a cell free extract using recombinant PO and a semi-purified MIPP enzyme made from cells over-expressing MIPP. Addition of PO inhibits MIPP activity, and can be reversed by inclusion of a PO inhibitor. How this is actually achieved is unclear. Western blotting shows no increase in mobility of the MIPP enzyme, indicating that it is not directly cleaved. As MIPP is prepared as a membrane fraction, it would seem likely that direct target of PO is a co-purified peptide, whose current identity is unknown. In whole cells, IP_3_ concentrations rise by 30 minutes and then drop to basal values within an hour. Both *in vitro* and *in vivo* assays therefore indicate a rapid change of MIPP activity following loss of PO activity.

## PO AND GENE REGULATION

Although tempting to assume that the rise in IP_3_ following PO inhibition directly leads to Li^+^ resistance, there are a number of observations that argue that this is not the case [35]. First, there is the theoretical argument that as Li^+^ is an uncompetitive inhibitor of IMPase, elevating the concentration of its inositol phosphate substrates should enhance rather than alleviate Li^+^ inhibition [39]. Only increased IMPase gene expression would confer Li^+^ resistance, as seen for Li^+^ inhibition of PIP_3_. Second, in contrast to DpoA, genetic manipulation of MIPP, either by gene disruption or over-expression, has no long-term direct effect on IP_3_ concentration, however both have Li^+^ hypersensitive phenotypes. This argues that whilst DpoA mediated changes in MIPP activity lead to transient changes in IP_3_ levels, it is not the end point of the signal pathway, but in fact lies en route to more long-term changes in cell behaviour.

Further investigation showed that PO inhibition leads to decreased expression of the genes encoding inositol synthesis (IMPase and Ino1) and inositol recycling (IPP and IP_3_ 5’phosphatase genes) [35]. Elevation of 5’phosphatase gene expression fits the observation that DpoA mutants has decreased 5’phosphatase activity [27], and increased IMPase expression explains how cells become Li^+^ resistant, as consistent with an uncompetitive mode of inhibition by increasing enzyme rather than substrate concentration. DpoA mediated gene expression is dependent on MIPP, as PO inhibitors no longer elevate gene expression in a MIPP mutant strain.

The exact mechanism leading to gene expression changes is not fully worked out, however there are some strong pointers to other elements of the signalling pathway [35]. Over-expression of MIPP1 both causes Li^+^ hypersensitivity and lowers concentrations of I(1,3,4,5,6)P_5 _and IP_6_. I(1,3,4,5,6)P_5_ has previously been shown to regulate* ino1* and *pho* gene expression in yeast [40], and has been found in mammalian cells to mediate some aspects of signalling *via *the extracellular protein ligand Wnt [41]. Consistent with the involvement of I(1,3,4,5,6)P_5_ or a related higher order inositol phosphate, over-expression of the IP_3_ kinases, inositol polyphosphate multikinase and inositol 1,3,4-triphosphate 5/6 kinase, which produce I(1,3,4,5,6)P_5_, elevate Ino1 and IMPase gene expression and confers Li^+^ resistance [35]. Furthermore, I(1,3,4,5,6)P_5_ and IP_6_ regulate *ino1* gene expression in yeast by regulation of chromatin re-modelling *via *the ATP-dependent re-modellers Swi/SNF2 and Ino80 [40, 42]. These observations suggest that DpoA and MIPP may in fact act within a gene regulatory network that also involves IP_5_ and IP_6_ signalling and chromatin remodelling.

## PO SIGNALLING IN MAMMALIAN CELLS

These *Dictyostelium* observations reveal the existence of a novel signalling pathway that is mediated *via *changes in PO and MIPP activity, however, is this a conserved pathway? A number of observations link PO activity to changes in IP signalling and Li^+^ sensitivity in mammalian systems.

In rats and humans, PO activity and expression has been found throughout the tissues of the body, but it is enriched in the brain. Looking at the brain in more detail, it is clear that there is differential enrichment within the brain, so that higher PO levels are seen in hippocampal CA1 pyramidal cells, cortical interneurons and pyramidal cells, striatal spiny neurons, cells in the thalamus and cerebllar Purkinje cells [43]. Interestingly, in many of these cells PO expression colocalizes with the IP_3_ receptor type 1 protein expression, suggesting a regulatory relationship between PO and IP_3_ signalling [44, 45].

In astroglioma cells, treatment with PO inhibitors or PO-specific siRNA elevates IP_3_ signalling in response to Substance P [46]. In primary neurons, Li^+^ mediated growth cone spreading is reversed by either addition of myo-inositol or PO inhibition [47]. Finally, in COS-7 cells, the cellular process of macro-autophagy and clearance of Huntingtin and α-synuclein protein aggregates is stimulated by Li^+^ through an inositol-depletion mechanism [48]. Again Li^+^ action on these processes is suppressed by PO inhibition.

In addition, PO inhibitor treatment of HEK293 cells leads to decreased expression of the human homologues of Ino1 (ISYNA1) and IMPase (IMPA1 and IMPA2). This gene regulatory effect is suppressed by MIPP1 specific siRNAs [35]. These results demonstrate a conserved relationship between PO activity and gene expression; it is currently unknown whether altered I(1,3,4,5,6)P_5_, IP_6_ or chromatin re-modelling is also required for this signalling pathway.

In *Dictyostelium*, DpoA expression and PO activity is developmentally regulated, being significantly up-regulated just prior to the formation of multicellularity [35]. It is not known what induces these changes. In contrast, recent results in SH-SY5Y neuroblastoma cells demonstrate PO expression and activity is up-regulated following retinoic acid induction of cell differentiation [49]. Interestingly, PO activity is also required for gene expression in this system. However in this case there is neither up-regulation of genes involved with inositol biosynthesis, nor long-term changes in IP_3_ concentration. This suggests that PO may also mediate gene regulation in alternative signalling pathways to that modulating inositol biosynthesis.

## BIPOLAR SUSCEPTIBILITY GENES AND ENVIRONMENTAL RISK

What insights do these observations offer into the origins of bipolar mood disorder? In addition to Li^+^, at least two other mood stabilizers, valproic acid (VPA) and carbamazepine (CBZ), alter neuronal cell behaviour in the same way as Li^+^, and again can be reversed by either addition of myo-inositol or PO inhibition [47]. Similarly, VPA and CBZ also induce macro-autophagy and protein aggregate clearance, and again these effects are reversed by PO inhibition [48]. The cellular relationship between mood stabilizers and PO therefore appears to reach beyond simply the effects of Li^+^, but has inositol phosphate signalling in common.

Altered PO activity has previously been linked to mental disorders. PO activity is decreased in schizophrenia patients and decreased in depression [50, 51]. In manic phase patients prior to treatment, plasma PO activity is decreased, but then reduced to below that of control patients following mood stabilizer treatment and bipolar patients [51]. An independent follow up study, which took into account possible influences of a second oligopeptidase known as ZIP, although failing to see a significant increase in schizophrenia patients, again observed a decrease in bipolar patients [52]. Reduced PO activity would be expected to lead to decreased IMPase expression, which could be suppressed by Li^+^ treatment.

The cause of decreased PO activity is unknown, however high doses of the cytokine IFN-α in patients undergoing immunotherapy have been associated with lowered serum PO activity; it has been suggested that this is associated with symptoms of depression [53]. Mood disorders have previously been associated with an altered the inflammatory response and bipolar disorder patients have altered expression of genes associated with the pro-inflammatory response [54].

Looking at the genes regulated by PO in HEK293 cells, it is notable that IMPA2 has previously been associated with bipolar mood disorder. Elevated IMPA2 expression has been reported in post-mortem patient brains [55]. This corresponds to the presence of a specific set of IMPA2 single nucleotide polymorphisms (SNPs) that associates with increased risk of developing bipolar disorder. These SNPs lie within the IMPA2 gene promoter and have been suggested result in decreased gene expression [55-57]; however further analysis is required to verify this link.

**Fig. (3):**
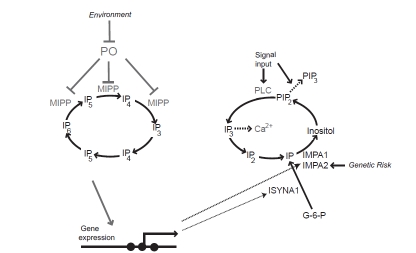
**A gene regulatory network modulates inositol phosphate signalling.** Human PO regulates expression of the IMPA1, IMPA2 and ISYNA1 genes *via* control of MIPP. This gene regulatory network is discrete from ligand-stimulated regulation of IP_3_ and PIP_3_ signalling, and modulates long-term changes in inositol phosphate signalling. Altered PO activity and IMPA2 gene expression have been observed in bipolar disorder patients, and may embody inputs from environmental and genetic risk factors respectively.

On the other hand, SNP analysis has failed to find a significant association of PO alleles and risk of bipolar disorder [58]. One interesting possibility is that PO lies at the intersection between environmental and genetic risk factors, with increased risk arising from inherited changes in gene promoter activity, such as suggested for IMPA2, in combination with environmentally induced changes in PO activity (Fig. **3**). One scenario, for example, could arise if an environmentally induced-response, perhaps *via *IFN-α, lead to decreased PO activity and increased IMPA1 and ISYNA1 gene expression. This would interact with aberrant regulation of the IMPA2 promoter to cause substantial elevation of inositol biosynthesis and over-active IP signalling, which in turn could be suppressed by Li^+^ or other mood stabilizers. Such enhanced signalling is seen in *Dictyostelium* when both the IMPase and Ino1 genes are over-expressed [35]. This causes abnormally high cell motility speed that is then suppressed by Li^+^.

## CONCLUSIONS

PO interacts with a novel intracellular signalling pathway, which regulates gene expression. This pathway requires the enzyme MIPP, which acts to regulate I(1,3,4,5,6)P_5_ and IP_6_, and regulates gene expression. IP_3_ is both decreased transiently following PO inhibition through decreased MIPP activity, and in the long term *via *decreased gene expression. This signalling pathway appears to be conserved in human cells, and has a number of characteristics that associate with observations on mental disorders. However, further work investigating the action of PO in model systems, as well as more genetic studies, will be required to establish the validity of these associations.

## ABBREVIATIONS

cAMP: = Cyclic adenosine monophosphate
DPG: = Diphosphoglycerate
GSK-3: = Glycogen synthase kinase-3
IMPase: = Inositol monophosphatase
Ino1: = Inositol synthase
IP: = Inositol phosphate
MIPP: = Multiple inositol polyphosphate phosphatase
PGM: = Phosphoglucomutase
PI: = Phosphatidyl inositol
PI(n,n,n)Px: = Phosphatidyl phosphate, where n = position of phosphate and x = number of phosphates
PI3K: = Phosphatidyl 3-kinase
PLC: = Phosopholipase C;
PO: = Prolyl oligopeptidase;
SNP: = Single nucleotide polymorphism
VPA: = Valproic acid
